# Full-Length Transcriptome: A Reliable Alternative for Single-Cell RNA-Seq Analysis in the Spleen of Teleost Without Reference Genome

**DOI:** 10.3389/fimmu.2021.737332

**Published:** 2021-09-27

**Authors:** Lixing Huang, Ying Qiao, Wei Xu, Linfeng Gong, Rongchao He, Weilu Qi, Qiancheng Gao, Hongyan Cai, Hans-Peter Grossart, Qingpi Yan

**Affiliations:** ^1^ Fisheries College, Key Laboratory of Healthy Mariculture for the East China Sea, Ministry of Agriculture, Jimei University, Xiamen, China; ^2^ Fourth Institute of Oceanography, Ministry of Natural Resources, Beihai, China; ^3^ Third Institute of Oceanography, Ministry of Natural Resources, Xiamen, China; ^4^ Department of Experimental Limnology, Leibniz Institute of Freshwater Ecology and Inland Fisheries, Stechlin, Germany; ^5^ Institute of Biochemistry and Biology, Postdam University, Potsdam, Germany

**Keywords:** scRNA-seq, full-length transcriptome, immune cell population, teleost, infection

## Abstract

Fish is considered as a supreme model for clarifying the evolution and regulatory mechanism of vertebrate immunity. However, the knowledge of distinct immune cell populations in fish is still limited, and further development of techniques advancing the identification of fish immune cell populations and their functions are required. Single cell RNA-seq (scRNA-seq) has provided a new approach for effective in-depth identification and characterization of cell subpopulations. Current approaches for scRNA-seq data analysis usually rely on comparison with a reference genome and hence are not suited for samples without any reference genome, which is currently very common in fish research. Here, we present an alternative, i.e. scRNA-seq data analysis with a full-length transcriptome as a reference, and evaluate this approach on samples from *Epinephelus coioides*-a teleost without any published genome. We show that it reconstructs well most of the present transcripts in the scRNA-seq data achieving a sensitivity equivalent to approaches relying on genome alignments of related species. Based on cell heterogeneity and known markers, we characterized four cell types: T cells, B cells, monocytes/macrophages (Mo/MΦ) and NCC (non-specific cytotoxic cells). Further analysis indicated the presence of two subsets of Mo/MΦ including M1 and M2 type, as well as four subsets in B cells, i.e. mature B cells, immature B cells, pre B cells and early-pre B cells. Our research will provide new clues for understanding biological characteristics, development and function of immune cell populations of teleost. Furthermore, our approach provides a reliable alternative for scRNA-seq data analysis in teleost for which no reference genome is currently available.

## Introduction

Fish provides an important human source of food and nutrition, and aquaculture plays a vital and increasing role in meeting the current global food demand (1, 2). The increasing harm of fish infection throughout the entire production chain requires more in-depth research on the molecular mechanisms of fish immune defense against pathogens (3–6). Moreover, fish represent the evolutionary oldest group to possess both innate and adaptive immunity (7–9). Therefore, fish are considered to be excellent models for elucidating in detail regulation mechanisms and evolution of the vertebrate immune system. Consequently, today, teleost fish constitutes the most widely used animal model of ectothermic immunity in immunological research (10–12).

However, several difficulties have emerged in the process of applying research results of mammalian immunology to fish immunology in recent years (13). One of the biggest obstacles stems from the lack of cell markers and molecular components of the immune system (13). As a result, although numerous fish immunomodulatory genes homologous to previously known mammals have been discovered and cloned in the past four decades, our understanding on “*in vitro*” functions of bony fish leukocytes remained greatly limited for quite a long time (13). In recent years, however, some *in vivo* and *in vitro* functional responses of fish lymphocytes have been reported (14). Currently, the number of fish lymphocyte-specific markers continues to increase, continuously facilitating the characterization of lymphocyte subpopulations (14). In order to explore the functional immunity of lymphocyte reactions, and raise the standard of fish immunology research, it will be necessary to combine the accumulated knowledge on immune gene products with the increasing number of molecular and cellular markers (14). Yet, a comprehensive and rapid identification approach of specific cell markers is urgently needed.

Latest progress of scRNA-seq provides a cost-effective method for obtaining extensive sets of single-cell-level transcriptomic data of different species and tissues. These data enable to account for cell heterogeneity, cell subtype-specific expression patterns and temporal changes in gene expression. This renders the scRNA-seq a highly powerful tool for identifying specific cell populations as well as new cell markers (15). Theoretically, the scRNA-seq technology allows for comprehensive classification of virtually every cell type on the planet (15). Consequently, scRNA-seq seems to be the perfect technique greatly advancing identification of fish immune cell populations and their functions. For example, Niu et al. (16) reported on the first scRNA-seq analysis of Nile tilapia (*Oreochromis niloticus*), an economically farmed fish species rather than a model organism, which revealed different subsets of non-specific cytotoxic cells in fish for the first time (16).

Without any doubt, the strength of scRNA-seq is its ability to recognize known, but also new, previously non-characterized cell types. To better achieve this, high-throughput data analysis is required to characterize the multitude of cell types detected by scRNA-seq from heterogeneous tissue samples. Characterization takes place in two steps: 1) grouping similar cells into non-overlapping clusters, and 2) labeling clusters, i.e. identifying cell types in each cluster. Yet, the current methods of reconstructing transcripts from these data mainly depend on alignment with known reference genomes, which hinders application to samples with incomplete reference genomes. There are numerous kinds of economic fish, whereby the outbred species usually display a high genetic variability in immune responses, but most of their genomes haven’t been sequenced or published, which currently is the biggest obstacle to research on the immune system of economic fish when using scRNA-seq. Hence, developing an universal solution for scRNA-seq transcriptome reconstruction for fish without any reference genome would provide a big advancement to improve current aquaculture measures.

In the present study, we carried out scRNA-seq analysis of the entire spleen from *Epinephelus coioides*-a teleost whose genome has not yet been published. Before sampling, the *E. coioides* was infected by *Pseudomonas plecoglossicida* for 2 days at 18°C. This model system is interesting because it shows the most intense pathogen-host interaction during the “visceral white spot disease”, which, in recent years, has developed high incidences in southern China (17–22). In order to verify our results globally, we used two different strategies: i) aligning the reads with the full-length transcriptome of *E. coioides* which we sequenced for the first time, and ii) aligning the reads with the reference genome of a closely related species-*Epinephelus lanceolatus* (PRJNA625542). The combination of full-length transcriptome sequencing and scRNA-seq represents a new cell classification computation strategy, which enables complete reconstruction of most transcripts present in the scRNA-seq data without *a priori* whole genome sequencing of the respective fish species. Yet, its sensitivity is equivalent to the traditional approach relying on whole genome alignments. Four cell types were identified: T cells, B cells, monocytes/macrophages (Mo/MΦ) and NCC. Further analysis proved the existing of populations, differentiated cells, and intermediates. Our research provides in depth understanding of biological characteristics, development and function of immune cell populations of teleost.

## Materials and Methods

### Artificial Infection

We randomly divided healthy *E. coioides* weighing 45.0 ± 1.5 g into groups of 10 fish. Each fish received 10^3^ CFU/g of the bacterial pathogen *P. plecoglossicida via* intraperitoneal injection, while sterile PBS was used as a negative control (23).

For scRNA-seq, after 48 h post-infection (hpi) with *P. plecoglossicida*, the spleen specimen from one *E. coioides* specimen was sampled (24). For full-length RNA-seq, spleen and intestines from three *E. coioides* were sampled at 0, 24, 48, 72 and 96 hpi.

Experiments involving animals were conducted in accordance with the “Guide for the Care and Use of Laboratory Animals” proposed by the National Institutes of Health. The protocol involved has been approved by the Animal Ethics Committee of Jimei University (JMULAC201159).

### Full-Length RNA-Seq

The full-length RNA-seq was done by Gene Denovo (Guangzhou, China). According to the manufacturer’s guideline, RNA from each tissue was extracted individually with Trizol reagent (Invitrogen, CA, USA) and 5 μg of RNA from each tissue were pooled for one library. The first and second strand cDNA were synthesized from polyA mRNA with Oligo-dT primers according to the manufacturer’s guideline (Clontech SMARTER cDNA synthesis kit). The size fractionation and selection (<4 kb and >4 kb) was carried out on the BluePippin™ Size Selection System (Sage Science, Beverly, MA). The Pacific Biosciences DNA Template Prep Kit 2.0 was used for the SMRT bell library construction, thereafter the Pacific Bioscience Sequel System was used for the SMRT sequencing. The SMRTlink (version 5.1) software was used for the circular consensus sequence (CCS) generation from initial sequence data (25). CCS was then identified as non-full-length and full-length reads according to poly(A) tails as well as 3’ and 5’ adapters. The Iterative Clustering for Error Correction (ICE) method was carried out to identify clusters of transcripts on the basis of reiterative assignment as well as pairwise alignment of full-length reads. Cluster consensus reads with non-full-length reads were polished by using the Arrow software to obtain high-quality isoforms. Full-length transcripts were annotated by searching against the Nr, UniProtKB and KOG databases with BLASTx (26). Function classifying was carried out by GO annotation, KEGG orthology and pathway annotations (27). The open reading frame (ORF) was determined for each full-length cDNA sequence *via* ANGLE (28). CPC (29), PLEK (30), Pfam-scan (31), and CNCI (32) analyses were performed for prediction of the long non-coding RNAs (lncRNAs). SUPPA2 was used to predict alternative splicing (33). Hmmscan was carried out to predict transcriptional factors. All raw sequencing data is stored in the NCBI Sequence Read Archive (SRA) under the accession number: SRP321375.

### scRNA-Seq

Library synthesis and scRNA-seq were done by Gene Denovo (Guangzhou, China). Lysis of blood cells in the spleen was performed with the Red Blood Cell Lysis Solution (Miltenyi Biotec, Germany). The remaining cells were diluted to a concentration of 1000 cells/μL (16). Barcode-labeling of these cells and subsequently mixed with reverse transcriptase into a Gel Beads-In-Emulsions (GEMs). The sequencing primers R1 and P5 arms were used for the cDNA library PCR amplification, and the two groups of cDNA libraries were pooled on the Illumina 10× Gemonics Chromium platform (10× GENOMICS). All raw sequencing data is stored in the NCBI Sequence Read Archive (SRA) under the accession number: SRP321375.

### scRNA-Seq Data Processing

Cell-Ranger (v2.0) data filtering, quantification, identification and comparison yielded the gene expression matrix of each cell. Further analysis of cell filtration, standardization, classification of cell subgroups, analysis of differentially expressed genes of different cell subgroups, and selection of marker genes were performed using the Seurat software (34). The reads were then aligned to the full-length transcriptome of *E. coioides* and the reference genome of a closely related species-*E. lanceolatus* (PRJNA625542) on the 10× Genomics website.

Cell Ranger was then used for data quality analysis of the raw data and then compared to the full-length transcriptome of *E. coioides* and the genome of *E. lanceolatus*. In our Illumina paired-end sequencing, Read 1 used a 16 bp GemCode barcode to distinguish different cells, and also included a 10 bp UMI (Unique Molecular Identifier); Read 2 was a cDNA sequence fragment. The STAR alignment software (35) was used to align Read 2 with the references. The GTF annotation identified the alignment results as introns, exons and intergenic regions. If the transcriptome reads matched only one specific gene, they were considered to be UMI-mapped and used for UMI counting.

The UMI corresponding to each gene id of each barcode was deduplicated using Cell Ranger, and the number of unique UMIs obtained was used as the expression level of cell genes. The expected number of cells is marked as N. The barcodes were arranged according to the UMI count from largest to smallest, and the first N barcodes were kept. The number of UMI counts for the 99^th^ percentile was recorded as m, the barcode was filtered for UMI count < 10% ^*^ m, and the obtained cells were valid cells.

The Seurat software (34) was used to normalize the expression level, which was then used for the Principal Component Analysis. The Seurat software clusters and groups the cells based on graph theory clustering algorithms. On the basis of the classification results of cell subgroups, the nonlinear tSNE clustering method was used for further visualization (36).

The likelihood-ratio test (37) was carried out to screen differentially expressed genes for a single cluster. Seurat’s bimod likelihood ratio statistical test was then carried out to analyze differences in gene expression level of different cell populations, and up-regulated genes of each population were picked out. Based on these steps, we analyzed the transcriptional regulation mode of a single cell subpopulation, and further screened the specific gene markers expressed by each subpopulation.

Genes that vary a lot are often highly informative for identifying cell subpopulations or ordering cells along a trajectory. For the pseudo-time analysis, we selected genes based on their variance, using the disp_table () and ordering _genes () programs to select those gene that mean expression >= 0.5 and dispersion empirical >= 1. The selected genes and their main functions were listed in **Supplementary Table 1**.

## Results

### Identifying the Full-Length Transcriptome of *P. plecoglossicida* Infected *E. coioides*

**Figure 1:**
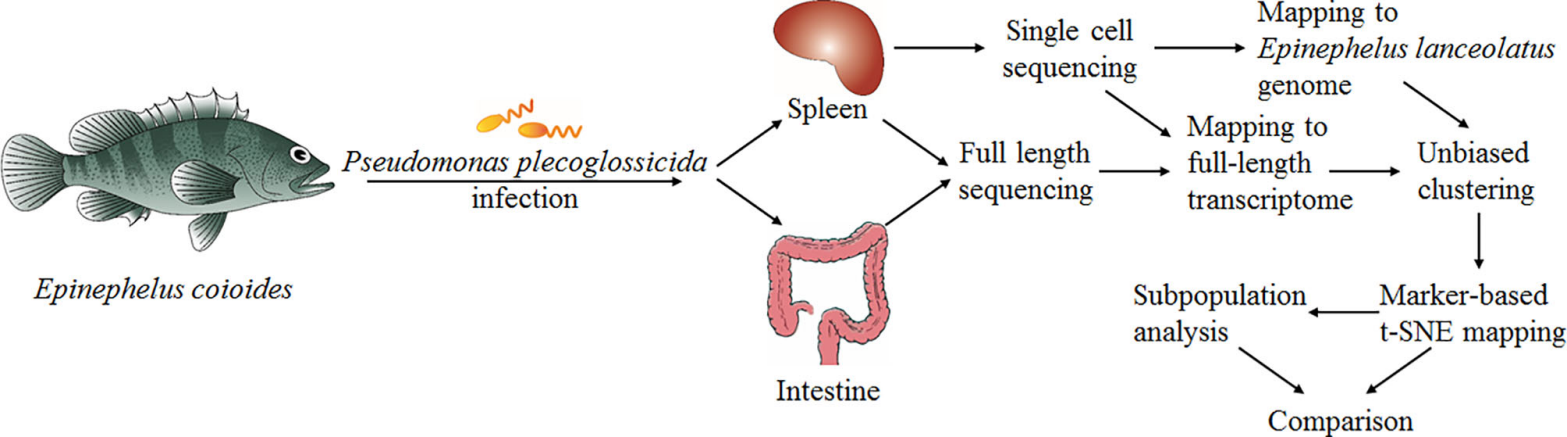
Overall work flow diagram of cell classification and single cell sequencing analysis.

**Figure 2:**
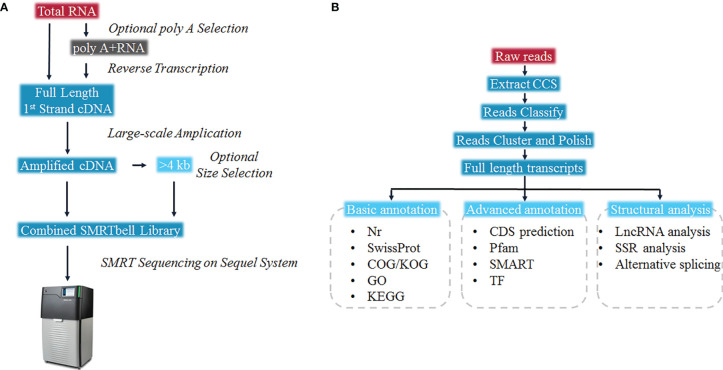
Overall work flow diagram of **(A)** Sequel full length transcriptome cDNA library construction, and **(B)** full-length transcriptome data analysis.

To present the possibility of scRNA-seq data analysis using the full-length transcriptome as a reference, and evaluate it for samples of the teleost *E. coioides* (whose genome has not yet been published), the full-length transcriptome of *E. coioides* was sequenced for the first time in the present study (**Figure 1**). To capture the diversity of transcript isoforms expressed during the *P. plecoglossicida* infection, we constructed the full-length RNA-seq library of spleen and intestine samples from 0 to 96 hpi (**Figure 2A**). According to the analysis process shown in **Figure 2B**, full-length transcripts of *E. coioides* were characterized.

**Figure 3:**
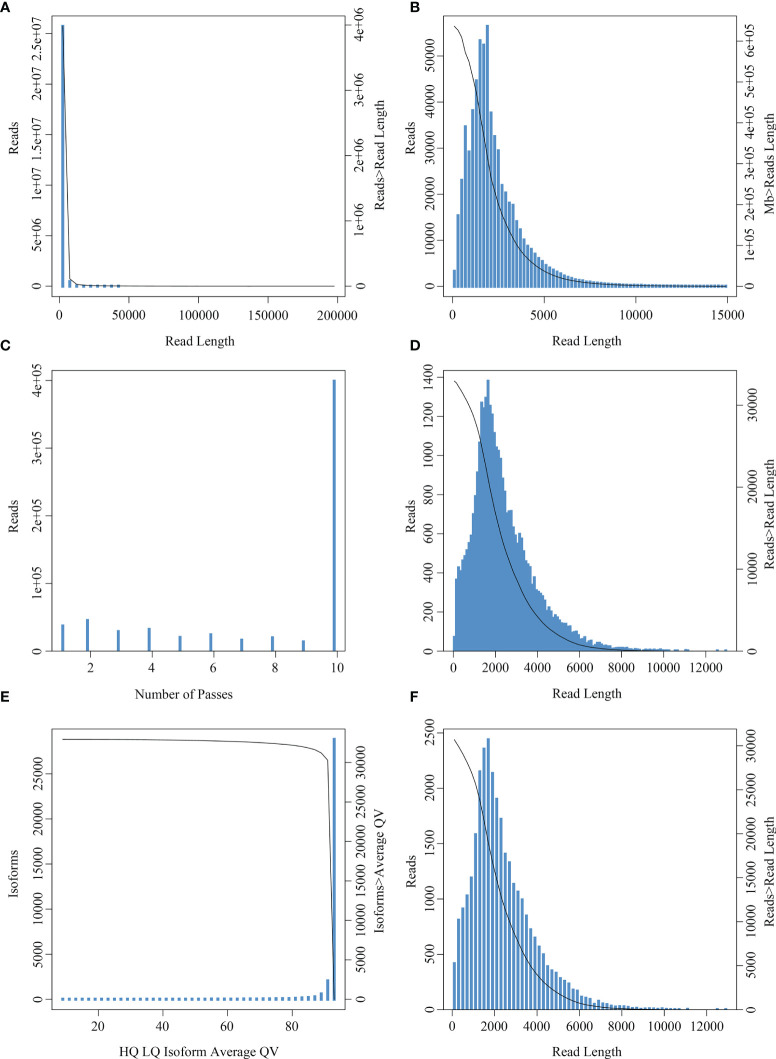
Read number and length distribution after full length transcriptomic sequencing. **(A)** Subread length distribution: the abscissa represents the length of subreads, and the ordinate is the number of subreads. **(B)** CCS length distribution: the x-axis represents the length of the reads, and the y-axis on the left represents the coordinates of the column graph, indicating the number of reads whose length is within a certain range (x-axis); the y-axis on the right is the coordinate of the graph, indicating the number of reads whose length is greater than a certain value (x-axis). **(C)** CCS passes distribution: the abscissa represents the number of full passes and the ordinate represents the number of CCS sequences with corresponding full passes. **(D)** Consistent sequence length distribution: the abscissa represents the length of the consistent sequence, the left ordinate represents the number of sequences with the length, and the right ordinate represents the number of sequences with the length greater than a certain value (x-axis). **(E)** Mean mass distribution map of consistency series: the abscissa represents the quality values of high- and low-quality sequences, and the ordinate represents the number of consistent sequences of the respective quality values. **(F)** Isoform length distribution: the x-axis represents the length of isoforms, and the y-axis on the left represents the coordinate of the column graph, representing the number of isoforms whose length is within a certain range (x-axis); the y-axis on the right is the coordinate of the graph, indicating the number of isoforms whose length is greater than a certain value (x-axis).

A total of 21,132,749 subreads with an average size of 1507 bp were obtained by filtering the Raw reads generated by sequencing (**Figure 3A**). A total of 1,075,793,700 circular consensus sequences (CCSs) were obtained after self-correction of all subreads (**Figures 3B, C**), which were then used to identify full-length non-chimeric reads. As a result, a total of 32,715 polished high-quality isoforms were identified (**Figures 3D, E**), and then redundancy of high-quality consistent sequences in each library was removed by Cd-hit-v4.6.7. Sequences with 99% similarity were combined to obtain the full-length transcriptome of each sample, with a total length of 72,588,100 bp (**Figure 3F**). The PacBio-SMRT technology used in this study improved our sequencing ability significantly and provided longer reads than the second-generation Illumina sequencing, which laid the foundation for current research on *E. coioides* full-length transcripts and subsequent single-cell sequencing.

### Full-Length Transcripts Annotations

**Figure 4:**
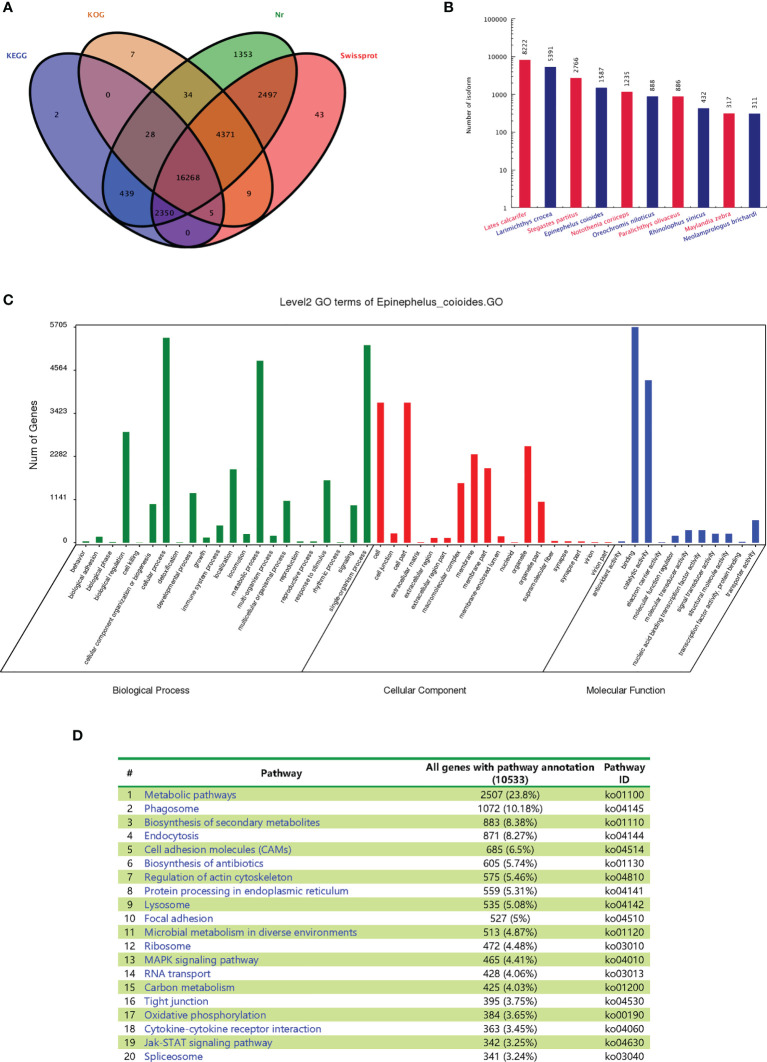
Functional annotations of the full-length transcripts with Nr, SwissProt, GO and KEGG. **(A)** Venn analysis of annotation results of four databases: Nr, Swiss Prot, KEGG and COG/KOG. **(B)** Statistical map of species distribution (only the top 10 species are shown): after comparing isoform sequences with the Nr database by BlastX, the sequence with the best (lowest E value) hit to the isoform in the Nr database was taken as the corresponding homologous sequence to determine homologous sequences of the species. The number of homologous sequences of each species was statistically compared. **(C)** Go function classification chart. **(D)** Distributions of the KEGG pathways.

All *E. coioides* full-length transcripts obtained above were aligned to KEGG, KOG, Nr and Swissprot. In total, 27,405 transcripts were successfully annotated and 3,334 transcripts failed. Among them, 16,268 transcripts were found in all databases. Specifically, 19,091, 20,721, 27,339 and 25,542 transcripts were annotated by KOG, KEGG, Nr and Swissprot, respectively (**Figure 4A**).

After BLASTx was used to compare the isoform sequences with the Nr database, the sequence with the best alignment result (lowest E value) of each isoform in the Nr database was selected as the corresponding homologous sequence. This allowed us to determine the respective homologous sequence, and the number of homologous sequences of each fish species was counted. The largest number of homologous sequences is not from *E. coioides* (1587), but *Lates calcarifer* (8222), *Larimichthys crocea* (5391) and *Stegastes partitus* (2766) (**Figure 4B**). The statistics of the results are restricted by the sequence information included in the Nr database. If the target species of interest has little entries, then the alignment will result in lower numbers of homologous sequences. As no whole genome of *E. coioides* has been published, there are fewer protein sequences in the database, which leads to the relatively lower number of observed homologous sequences for *E. coioides*.

The main Gene Ontology (GO) annotations distribution was shown in **Figure 4C**. “Cellular process”, “metabolic process” and “single organism process” were the most abundant sub-categories of the identified biological processes. “Cell”, “cell part”, “organelle” and “membrane” were the most abundant sub-categories of cellular components. “Binding” and “catalytic activity” were the most abundant sub-categories of molecular functions. The major distribution of the KEGG annotations was enriched in “metabolic pathways”, “phagosome”, and “biosynthesis of secondary metabolites” (**Figure 4D**).

**Figure 5:**
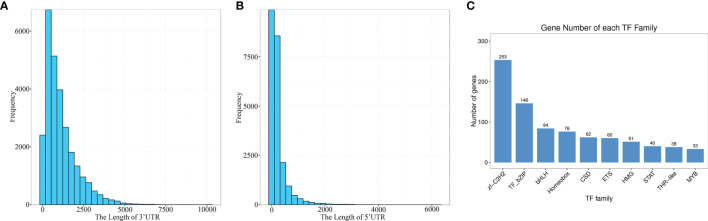
Prediction of the coding sequences and transcription factors. **(A)** The length of 3’ UTR. **(B)** The length of 5’ UTR. **(C)** TF family distribution (top 10).

CDS (coding sequence) refers to a sequence encoding a protein product, which corresponds to the codon of a protein. BLASTx was used to align the isomers with NR, Swiss prot, KEGG and COG/KOG in order. The highest ranked protein in the alignment result was selected as the isoform coding region sequence. Translation into the amino acid sequence according to the standard codon table (38) allowed to derive the nucleotide sequence (5 ‘ to 3’) as well as the amino acid sequence of the isoform coding region. Finally, for isoforms which could not be aligned using the above protein libraries, their coding regions were predicted with ANGEL (https://github.com/PacificBiosciences/ANGEL) to generate the nucleic acid sequence (5 ‘ to 3’) as well as the amino acid sequence of the coding region. In total, 27,558 CDS were obtained in this study. The 3 ‘and 5’ UTR length distributions are given in **Figures 5A, B**.

The predicted CDS were then aligned with the Animal TFDB 2.0 database using hmmscan to identify transcription factors (TFs). A total of 1209 TFs were identified, which were categorized into 59 families, and the top ten were zf-C2H2, TF-bZIP, bHLH, Homeobox, CSD, ETS, HMG, STAT, THR-like and MYB (**Figure 5C**). These results provided a meaningful basis for the future research on TFs and gene expression regulation in *E. coioides*.

In addition, the lncRNAs and alternative splicing (AS) events were predicted. A total of 3,000 lncRNAs were identified based on the full-length transcripts failed to align to NR, Swiss prot, KEGG as well as COG/KOG using CNCI and CPC approaches (**Supplementary Figure 1A**). In total, 2,468 AS events were identified in the transcripts of *E. coioides* (**Supplementary Figure 1B**). In this study, the proportions of the main AS events were as follows: retained intron (42.59%), alternative 3’ splice site (21.23%), alternative 5’ splice site (24.55%), skipped exon (5.71%), alternative first exon (1.82%), alternative last exon (0.12%), and mutually exclusive exon (3.97%) (**Supplementary Figure 1B**).

### Sequencing Saturation and Data Utilization Comparison With Different References

The scRNA-seq obtained 403,326,770 reads. Among them, 97.60% of the reads owned valid barcodes (**Table 1**). Q30 Bases in barcode, RNA read and UMI accounted for 95.40%, 91.20% and 95.10%. When full-length transcriptome and the *E. lanceolatus* genome were used as reference for data analysis, the sequencing saturation was slightly different, which can be related to the different gene information contained in these two references. In general, however, their sequencing saturations were very close, which proved that the gene information provided by the full-length transcriptome was enough to support the subsequent analysis.

**Table 1:** Sequencing saturation comparison between different references.

Reference	Number of Reads	Valid Barcodes	Sequencing Saturation	Q30 Bases in Barcode	Q30 Bases in RNA Read	Q30 Bases in UMI
Full-length transcriptome	403,326,770	97.60%	81.80%	95.40%	91.20%	95.10%
*Epinephelus lanceolatus*			84.10%			

Data utilization comparison of using these two references was carried out (**Table 2**), which revealed that the number of cells detected did not differ much, while the amount of genes detected for each cell type was equal. The obvious difference lies in the reads mapped confidently to the genome and those mapped confidently to the transcriptome. The proportion of “reads mapped confidently to genome” was 84.4% when using *E. lanceolatus* genome as reference, and 51.4% when using the full-length transcriptome as reference. The proportion of “reads mapped confidently to transcriptome” was 68% with *E. lanceolatus* genome as reference, and 50% with full-length transcriptome as reference. These results indicate a higher utilization rate of data when using the genome of *E. lanceolatus* as the reference, yet, a substantial amount of reads can be confidentially mapped when using the full-length transcriptome as a reference.

**Table 2:** Data utilization comparison between different references.

Reference	Full-length transcriptome	*Epinephelus Lanceolatus*
Estimated Number of Cells	3,239	3,455
Fraction Reads in Cells	67.40%	68.00%
Mean Reads per Cell	124,522	116,737
Median Genes per Cell	1,550	1,504
Total Genes Detected	12,725	17,270
Median UMI Counts per Cell	5,227	6,249
Reads Mapped Confidently to Genome	51.40%	84.40%
Reads Mapped Confidently to Intergenic Regions	0.00%	8.00%
Reads Mapped Confidently to Intronic Regions	0.00%	5.00%
Reads Mapped Confidently to Exonic Regions	51.40%	71.50%
Reads Mapped Confidently to Transcriptome	50.00%	68.00%

### Cells Clustering Comparison With Different Rerences

**Figure 6:**
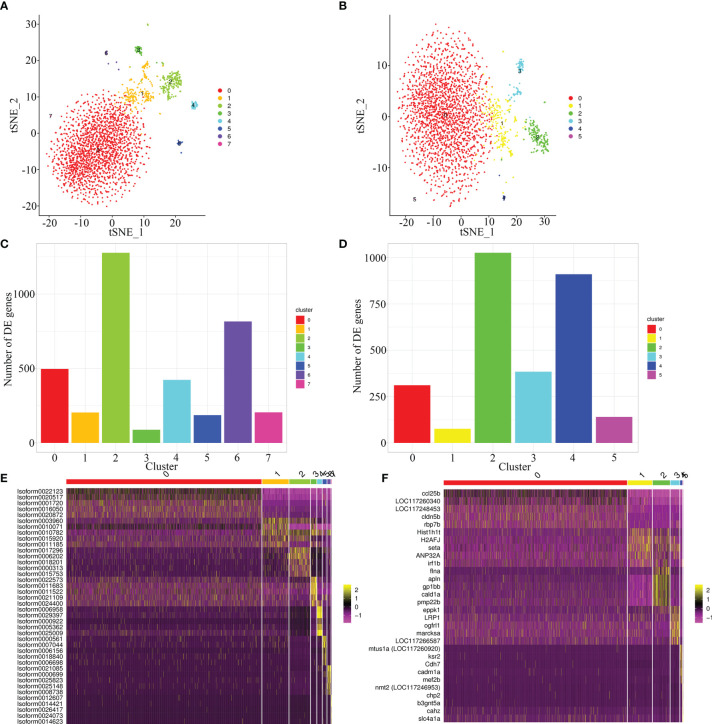
Cell sorting based on full-length transcriptome and *E*. *lanceolatus* genome. **(A, B)** The tSNE nonlinear clustering was used to visualize the classification results of *E*. *coioides* spleen cell populations based on full-length transcriptome **(A)** and the whole *E. lanceolatus* genome **(B)**. **(C, D)** Statistical histogram of the number of up-regulated genes in each sub-cluster based on full-length transcriptome **(C)** and *E. lanceolatus* genome **(D)**. **(E, F)** Heatmap of the top 5 up-regulated expression genes from each cluster as a marker gene based on full-length transcriptome **(E)** and *E. lanceolatus* genome **(F)**. Each column in the figure represents a cell, and each row represents a gene. The expression levels of genes in different cells are indicated by different colors. The more yellow, the higher the expression level, and the more purple, the lower the expression level.

To compare the quality of cells clustering based on the full-length transcriptome and *E. lanceolatus* genome, the unsupervised cluster detection algorithm (SEURAT) was applied to group cells based on the similarity of gene expression. With the full-length transcriptome as the reference, 7 cell clusters were identified (**Figure 6A**). With *E. lanceolatus* genome as the reference, 5 cell clusters were identified (**Figure 6B**). From the cell clustering results, the accuracy of cells clustering based on the full-length transcriptome was somewhat higher, but the difference was not particularly obvious. Moreover, 75% of the cells were divided into a subgroup, so it was not easy to evaluate which result was better based on the respective analysis. Therefore, we further mapped the cell clustering results based on the genome of *E. lanceolatus* back to the results based on the full-length transcriptome, to check for the consistency of both strategies. According to the mapping analysis (**Supplementary Figure 2**), the results of both cell clustering approaches were relatively consistent. Further, the highly expressed genes were identified (**Figures 6C, D** and **Supplementary Table 1**), which enabled us to screen the 5 genes with the highest expression level in each cluster and built a heat map. The obtained results show that each cluster had a clear separation boundary (**Figures 6E, F**), supporting the accuracy of cells clustering based on both references.

At the same time, we also used UMAP, the latest dimension reduction method of scRNA-seq, to proof for the results of cell clustering (**Supplementary Figure 3**). Our results revealed that the cell clustering based on the full-length transcriptome was indeed better: cell clustering was more concentrated (the cells were more aggregately distributed in the cell clusters), and the differentiation between cell clusters was more obvious. We speculated that even if the alignment rate of the *E. lanceolatus* genome was higher, there must be differences between the genome sequences of the *E. lanceolatus* and the *E. coioides*, which would inevitably lead to the inaccuracy of the alignment data and the inaccuracy of the clustering. However, genome alignment of the 10× scRNA-seq only uses the 96 bp sequence of the 3’ end of mRNA for alignment and clustering analysis, consequently the original discrimination is not pronounced. Therefore, when using the *E. lanceolatus* genome as a reference, it is likely that the mRNA sequences of different genes in the *E. coioides* were aligned to the same gene of the *E. lanceolatus* genome, which would reduce the difference between cells in a disguised form and result in an indistinguishable differentiation of cell groups. This problem doesn’t exist when using the full-length transcriptome as the reference for cell clustering analysis, because all sequences stem from *E. coioides* itself.

**Figure 7:**
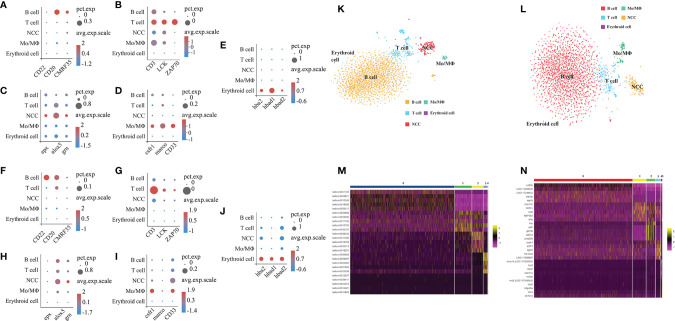
Categorization of cell types based on full-length transcriptome and the whole *E. lanceolatus* genome. Bubble plots of expression of the 5 marker cell types in all clusters based on full-length transcriptome **(A–E)** and *E. lanceolatus* genome **(F–J)**. X-axis depicts the name of the marker gene and Y-axis the name of the cell subpopulation; the size of the bubble represents the ratio of the sum of the expression of the marker gene in a certain subgroup to the sum of its total expression (all cells); the color of the bubble represents the average expression abundance of the marker gene in the cell subgroup; the more red the bubble color, the higher the average expression of the marker gene in the respective subgroup. **(K, L)** Identification of 5 cell subpopulations based on marker molecules. The results based on full-length transcriptome and *E. lanceolatus* genome are displayed in **(K, L)**, respectively. The 5 cell populations are represented by different colors (B cell: orange, T cell: blue, Mo/MΦ: green, NCC: red). **(M, N)** The heatmap of the top 5 up-regulated expression genes from each cell subpopulation as a marker gene based on the full-length transcriptome **(M)** and *E. lanceolatus* genome **(N)**.

To characterize the clusters based on the full-length transcriptome and the *E. lanceolatus* genome, we analyzed the genes’ expression in the clusters and inferred their putative identities from known markers (**Figures 7A–J**). For the analysis based on the full-length transcriptome, the clusters 0 and 5 expressing *CD22* (39), *CD20* (40) and *CMRF35* (41) belonged to the B cell population, the clusters 1 and 3 with the expressions of *CD3* (42, 43), *LCK* (44) and *ZAP70* (45) were clustered into the T cell population, the clusters 4 and 6 expressing *csfr1* (46), *marco* (47) and *CD33* (48) belonged to the Mo/MΦ population, cluster 2 with the expressions of *epx* (16), *alox5* (16) and *grn* (16, 49) belonged to the NCC population, and the erythroid cell population (cluster 7) was confirmed by *hba2*, *hbad1* and *hbad2* (50). Taken together, our analysis based on the full-length transcriptome successfully divided all clusters into 5 cell populations: T cells, B cells, Mo/MΦ, NCC and erythroid cells (**Figure 7K**). The analysis based on the *E. lanceolatus* genome also successfully divided the clusters into the same 5 cell populations using the same cell markers (**Figure 7L**). Thus, there was no significant difference in the proportion of different cell types between both analysis strategies. It is worth noting that the proportion of red blood cells is extremely low, indicating that the lysis of blood cells was optimal in the process of sample preparation. To further verify our classification results based on the full-length transcriptome, we tested the top 5 up-regulated genes in the 5 cell populations as well as performed a pseudo-time analysis (**Figures 7M, N**). Genes from the 5 cell populations clustered well in the heat map (**Figures 7M, N**), and they were distinct from each other. Taken together, our results confirm the accuracy of the full-length transcriptome strategy in cell clustering and classification.

### Cell Sub-Clustering and Identification Based on the Full-Length Transcriptome

**Figure 8:**
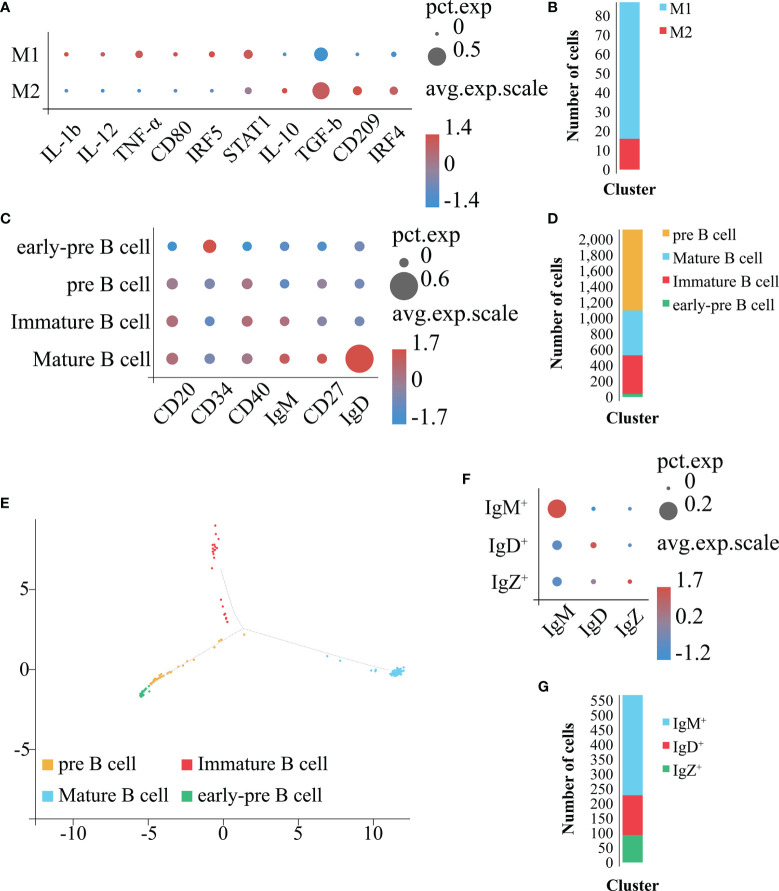
Identification of Mo/MΦ and B cell subpopulations. **(A)** Bubble plot refers to molecular expression marker in Mo/MΦ clusters. X-axis is the name of the marker gene and Y-axis the name of the Mo/MΦ subpopulation; the size of the bubble represents the ratio of the sum of the marker gene expression in a certain subpopulation to the sum of its expression in all Mo/MΦ cells; the color of the bubble represents the average expression abundance of the marker gene in the Mo/MΦ subpopulation; the more red the bubble, the higher the average expression level of the marker gene in the Mo/MΦ subpopulation. **(B)** Identification of the 2 Mo/MΦ subpopulations based on marker molecules. Cell subpopulations are represented by different colors (M1 type Mo/MΦ: blue, M2 type Mo/MΦ: red). **(C)** Bubble plot refers to molecular expression marker in all B cell clusters. X-axis is the name of the marker gene and Y-axis the name of the B cell subpopulation; the size of the bubble represents the ratio of the sum of the marker gene expression in a certain B cell subpopulation to the sum of its expression in all B cells; the color of the bubble represents the average expression abundance of the marker gene in the B cell subpopulations; the more red the bubble, the higher the average expression level of the marker gene in the B cell subpopulation. **(D)** Identification of 4 B cell subpopulations based on the identified marker genes. The 4 cell subpopulations are represented by different colors (Mature-B cell: blue, pre B cell: orange, Immature-B cell: red, early-pre B cell: green). **(E)** Pseudo-time analysis of B cell subpopulations. Each subpanel corresponds to previously identified subpopulations as shown in **(D)**. **(F)** Bubble plot refers to molecular expression marker in mature B cell clusters. X-axis is the name of the marker gene and Y-axis the name of the mature B cell subpopulation; the size of the bubble represents the ratio of the sum of the marker gene expression in a certain mature B cell subpopulation to the sum of its expression in all mature B cells; the color of the bubble represents the average expression abundance of the marker gene in the mature B cell subpopulations; the more red the bubble, the higher the average expression level of the marker gene in the mature B cell subpopulation. **(G)** Identification of 3 mature B cell subpopulations based on the identified marker genes. The 3 cell subpopulations are represented by different colors (IgM^+^: blue, IgD^+^: red, IgZ^+^: green).

In order to obtain a more detailed clustering and cell classification of Mo/MΦ (**Figures 8A, B**), we used the above mentioned approaches to analyze the Mo/MΦ population identified in **Figure 7K**. As a result, the Mo/MΦ population was further sub-divided into 2 clusters: 1) the cluster with the high expression level of *IL-1b*, *IL-12, TNF-α, CD80*, *IRF5* and *STAT1* belonged to the M1 type Mo/MΦ population, 2) the cluster with the high expression level of *IL-10*, *TGF-b*, *CD209* and *IRF4* belonged to the M2 type Mo/MΦ population (**Figures 8A, B**). Therefore, the 2 Mo/MΦ subgroups including M1 and M2 type Mo/MΦ are confirmed to exist in *E. coioides* spleen.

In order to obtain a more detailed clustering and cell classification of the B cells (**Figures 8C–G**), we used the above mentioned approaches to analyze the B cell population (**Figure 7K**). As a result, the B cell population was further sub-divided into 4 clusters: 1) the cluster with high expression level of *CD34* (51) and hardly expression level of *CD20* (40), *CD40* (52, 53), *IgM* (54), *CD27* (54) and *IgD* (55) were identified as early-pre B cell; 2) the cluster with expression of *CD20* and *CD40*, together with hardly expression of *CD34*, *IgM*, *CD27* and *IgD* were identified as pre B cell; 3) the cluster with expression of *CD20*, *CD40* and *IgM*, together with hardly expression level of *CD34*, *CD27* and *IgD* were identified as immature B cell; the cluster with the highest expression level of *CD20*, *CD40*, *IgM*, *CD27* and *IgD*, together with hardly expression level of *CD34* were identified as mature B cell, respectively (**Figures 8C, D**). To verify the reliability of the classification results, we carried out a pseudo-time analysis (**Figure 8E**). The obtained results display a beginning with the early-pre B cells, followed by the pre B cells, and a divergence into two fates, the immature B cells and mature B cells, respectively (**Figure 8E**). Therefore, the 4 B cell subsets including early-pre B cell, pre B cell, mature and immature B cell are confirmed to exist in *E. coioides* spleen. Further analysis on the mature B cells sub-classified them into 3 clusters: the IgM^+^, IgD^+^ and IgZ^+^ highly expressing *IgM*, *IgD* and *IgZ*, respectively (**Figures 8F, G**). Hence, IgM^+^, IgD^+^ and IgZ^+^ are confirmed to exist in *E. coioides* spleen. Due to the lack of the above-mentioned specific antibodies for predicting cell markers, it was not possible to further characterize and test for the function of these cells at this time.

## Discussion

Although we have a deep understanding of fish immunology at the molecular level, *in vitro* and *in vivo* research on immune activity of teleost is still in its infancy. Due to the anatomical homology between vertebrates, there is an increasing interest in studying teleost physiology. At present, for specific fish leukocyte populations and subpopulations, the availability of cellular and determinant markers is very limited. This is a major and long-standing challenge faced by fish immunologists. In order to overcome this issue, many advanced technologies are applied to investigate functional immunity of teleost’s lymphocyte responses, including scRNA-seq. Yet, the existing scRNA-seq data analysis strategies rely heavily on *a priori* genome alignment, which constitutes the biggest obstacle for many economic fish for which no whole genome sequence is available. Hence, seeking for an alternative for reliable scRNA-seq transcriptome reconstruction for species without any reference genome is desirable.

In the present study, we proofed the power of scRNA-seq data analysis using the full-length transcriptome instead of a whole genome sequence as a reference by evaluating it for *E. coioides*-a teleost samples. We sequenced the full-length transcriptome of *E. coioides* for the first time, with a total length of 72,588,100 bp. In total, 27,405 transcripts were successfully annotated and 3,334 transcripts failed. Among them, 16,268 transcripts were found in all databases. Specifically, 19,091, 20,721, 27,339 and 25,542 transcripts were annotated by KOG, KEGG, Nr and Swissprot, respectively. The PacBio-SMRT technology used in this study improved our sequencing ability significantly and provided longer reads than the second-generation Illumina sequencing, which laid the foundation for current research on *E. coioides* full-length transcripts and subsequent single-cell sequencing.

Our approach enabled us to completely reconstructed most of the transcripts present in the scRNA-seq data set, whereby the sensitivity was equivalent to the approach using genome alignments of a closely related species. At the same time, the UMAP showed that cell clustering based on the full-length transcriptome performed better, i.e. cell clustering was more pronounced, and the differentiation between cell clusters was improved. Although the alignment rate to the *E. lanceolatus* genome was higher, there seem to be pronounced differences between the genome sequences of both *E. coioides* and its close relative *E. lanceolatus.* These would inevitably lead to the inaccuracy in sequence alignment and thus in clustering. Genome alignment of the 10× scRNA-seq, however, only uses the 96 bp sequence of the 3’ end of mRNA for alignment and clustering analysis, and the original discrimination is not high. Therefore, when using the *E. lanceolatus* genome as a reference, it is likely that the mRNA sequences of different genes in *E. coioides* align to the same gene of the *E. lanceolatus* genome. This would reduce the detected difference between cells in a disguised form, reducing the ability to differentiate between distinct cell types. This problem can be circumvented by using the full-length transcriptome as a reference for cell clustering as all sequences originate from *E. coioides* itself. Thus, our approach provides a reliable alternative for scRNA-seq data analysis in teleost without any reference whole genome.

We found that *E. coioides* samples comprised the complete library of B cells, T cells, NCC and Mo/MΦ, which is consistent with previous reports for zebrafish (56) and *O. niloticus* (16). Further analysis indicated the presence of two subsets of Mo/MΦ including M1 and M2 type, as well as four subsets in B cells, i.e. mature B cells, immature B cells, pre B cells and early-pre B cells.

Macrophages are key leukocytes for innate and adaptive immune responses (57). Macrophages are usually classified according to their polarization rather than their tissue location (58). The M1 type macrophages are typically activated and polarized by IFN-γ signaling. They produce the pro-inflammatory microenvironment by secreting inflammatory cytokines, and eliminate intracellular pathogens through the action of nitric oxide and reactive oxygen species (59, 60). Presence of M1 macrophages in the infected area indicates that macrophage polarization have occurred by sensing danger signals (61, 62). M1 macrophages are a common phenotype of phagocytes during a cell-mediated immune response (63). On the other hand, M2 macrophages have anti-inflammatory effects and play a central role in wound healing and tissue repair (64, 65). M2 macrophages can be activated by anti-inflammatory cytokines (IL-4 or IL-13) (66), and their main function is to produce extracellular matrix and polyamines to promote cell growth and division, as well as synthesis of proteins required for the healing process (67). The classical polarization of teleost macrophages after stimulation with LPS, IFN-γ + LPS, *Trypanosoma borreli* or zymosan parasites has been reported, while alternative polarization was observed after treatment with cAMP, prostaglandin E2 and IL-4/13 (68–75). In the present study, the clusters expressing *csfr1* (46), *marco* (47) and *CD33* (48) were identified as the Mo/MΦ population. *csfr1* is the encoding gene of colony-stimulating factor 1 receptor, which is an important regulator of Mo/MΦ in many fish (46), and considered as a specific surface marker of fish Mo/MΦ. *marco*, the encoding gene of macrophage receptor with collagenous structure, plays important roles in phagocytic cell-mediated innate immune responses (47). *marco* plays key regulatory roles in the bacterial binding, clearance, as well as polarization processes of teleost Mo/MΦ (47). CD33 is a sialoadhesin molecule and a member of the immunoglobulin supergene family, which is considered to be an excellent Mo/MΦ marker (48). Furthermore, the Mo/MΦ population were sub-divided into M1 and M2 type Mo/MΦ with M1 specific markers (*IL-1b*, *IL-12,TNF-α, CD80*, *IRF5* and *STAT1*) and M2 specific markers (*IL-10*, *TGF-b*, *CD209* and *IRF4*) in the present study. M1 macrophages produce pro-inflammatory cytokines including IL-1b, IL-12, and TNF-α (76). Through these, the pro-inflammatory cascade is activated, which leads to the elimination of pathogens (77). Vital transcription factors involved in the classical macrophage polarization are IFN regulatory factor 5 (IRF5) and signal transducer and activator of transcription 1 (STAT1), which activate the expression of pro-inflammatory mediators such as *IL-12* and *TNF-α*, and then enhance the biocidal function of macrophages (78). M2 macrophages produce IL-10 and transforming growth factor-b (TGF-b) and are associated with immune suppression and tissue remodeling (76). IFN regulatory factor 4 (IRF4) is one of the pivotal transcription factors involved in alternative polarization, whose activation leads to increased expression of CD209 (78). Hence, in the present study, we reasoned that the 2 Mo/MΦ subgroups including M1 and M2 type Mo/MΦ are confirmed to exist in *E. coioides* spleen.

B cells play a key role in the adaptive immune response of vertebrate humoral immunity. The development of mammalian B cells mainly occurs in the bone marrow (79). B cell development includes all the early stages of differentiation without antigen interaction, until maturity, antigen interaction, and finally antibody synthesis (80). B cells present different molecules at different stages of development/maturation and activation (81). Pro B cells arise after obligatory stimulation by the transcription factor PAX-5, which produces CD19. These CD34^+^ CD19^+^ CD10^+^ CD38^+^ cell surface expressing and TdT^+^ nuclear expressing cells lack pre B cell receptors or surface immunoglobulins, and characteristically initiate VDJ heavy chain rearrangement independent of any antigen exposure. Pro B cells were then differentiated into pre-B cells expressing CD34^−^ CD19^+^ CD10^+^ CD38^+^ TdT^−^ to obtain cytoplasm, and then surface mu heavy chains complexed with a transient surrogate immunoglobulin light chain. Immediately afterwards, a CD19^+^ CD10dim/^−^ CD38dim/^−^ immature B cell expresses surface IgM^+^ and physiologic light chain. Finally, CD19^+^ CD20^+^ B cells that co-express IgM and IgD heavy chains as well as lack the early differentiation markers CD34, CD10, CD38 or TdT will leave the bone marrow as transitional B cells and return to secondary lymphatic organs as fully mature B cells (82, 83).

Compared with mammals, the early B cell differentiation and diversification of antibody pools in birds do not occur in the bone marrow, but in a special gut related lymphoid tissue, the bursa of Fabricius (84). During embryonic development, B cell precursors migrate to the bursa primordium, where they proliferate and diversify the B cell receptor pool (85). Around hatch, these diverse B cells begin to migrate from the bursa of Fabricius to the peripheral lymphoid organs, but little is known about the regulation of the migration process (86). Masteller et al. proved that changes in cell surface glycosylation may be related to the colonization of the bursa of Fabricius (84). Pre-bursal and early B cells express the carbohydrate epitope sialyl Lewis(x) (CD15s), which is a carbohydrate moiety that participates in the adhesion of leukocytes to endothelial cells through selectin binding (84). In contrast, the B cells in the bursa of Fabricius were transformed into high-level expression of Lewis(x) (CD15) (87) after gene conversion. Interestingly, the loss of CD15 around ED15 is related to the time when developing B cells lose the ability to colonize the bursa of Fabricius (88). Around hatch, the surface glycosylation changed for the second time, and B cells down-regulated CD15 to low to moderate expression. Since peripheral B cells are also low in CD15, it seems likely that cells with low CD15 can leave the bursa (89, 90). Little is known about the expression of selectin E, P and O or other CD15/CD15s receptors in the bursa of Fabricius, but the reported time-limited expression patterns strongly indicate the contribution of the molecules to the bursal immigration and emigration.

B cells in fish are a kind of functional antibody-secreting cells, which are capable of producing specific antibodies to deal with certain invading foreign antigens and play an important role in adaptive immunity (91). Unlike mammals, there is no specific antibody that can accurately distinguish the development/differentiation state of teleost B cells, which hinders further research on the effect of its phagocytic function. However, detecting the expression levels of B-cell specific genes can provide a comparative method for studying the development of B-cells in teleost (92, 93). In the present study, the clusters expressing *CD22* (39), *CD20* (40) and *CMRF35* (41) were identified as the B cell population. CD22 can not only provide co-stimulatory signals for the activation of IgM^+^ B cells, but also play an important regulatory role in the macropinocytosis-dependent pathway that teleost IgM^+^ B cells mainly rely on to internalize large particles (94, 95). CD20 is a universal B cell marker, which is expressed by most B cells from the late pre-B lymphocytes, and is closely related to immune regulation and pro-inflammatory activity (40). CMRF35 is a B cell surface protein as well as an immunoregulatory molecule, which has been observed involving in the mucosal immunity of teleost (96). Then, the B cell population was further sub-divided into 4 clusters: 1) the cluster with high expression level of *CD34* (51) and hardly expression level of *CD20* (40), *CD40* (52, 53), *IgM* (54), *CD27* (54) and *IgD* (55) were identified as early-pre B cell; 2) the cluster with high expression level of *CD20* and *CD40*, together with hardly expression level of *CD34*, *IgM*, *CD27* and *IgD* were identified as pre B cell; 3) the cluster with high expression level of *CD20*, *CD40* and *IgM*, together with hardly expression level of *CD34*, *CD27* and *IgD* were identified as immature B cell; the cluster with the highest expression level of *CD20*, *CD40*, *IgM*, *CD27* and *IgD*, together with hardly expression level of *CD34* were identified as mature B cell, respectively. The CD34 molecule is a highly glycosylated type I transmembrane glycoprotein that is selectively expressed on the surface of human and other mammalian pro B cells, and gradually weakens to disappear as the cells mature (51). More and more research results have shown that CD34 molecules play an important role in mediating cell adhesion (51). CD40 regulates the activation, germinal center formation, antigen presentation and antibody production in B cells (52, 53). CD27 is a TNF receptor that stimulates B cells and promotes their differentiation after being activated by the TNF ligand CD70, thereby promoting anti-infection immunity. IgM, IgD and IgZ (also named as IgT) are the only immunoglobulin classes identified in fish (97, 98). IgM is considered to be a ubiquitous vertebrate immunoglobulin, and is the first characterized teleost immunoglobulin (99, 100). IgZ is a unique teleost immunoglobulin analogous to mammalian IgA (101–103), and proved to be involved in mucosal immunity (104–107). Compared with IgM and IgT, teleost IgD functions are less investigated. However, in channel catfish (*Ictalurus punctatus*), IgD is considered to be a mediator of innate immunity (108). Two populations of B cells were originally described in rainbow trout: one subpopulation expresses both IgM and IgD, and the other subpopulation only expresses IgT (107). Recently a third subpopulation expressing only IgD was also identified in trout (109). Therefore, 4 B cell subsets including early-pre B cell, pre B cell, mature and immature B cell are confirmed to exist in *E. coioides* spleen in the present study. Further analysis on the mature B cells sub-classified them into 3 clusters: the IgM^+^, IgD^+^ and IgZ^+^ highly expressing *IgM*, *IgD* and *IgZ*, respectively. Hence, IgM^+^, IgD^+^ and IgZ^+^ are confirmed to exist in *E. coioides* spleen.

T cells are white blood cells that work with macrophages to fight infection. Two types of T cells are confirmed for mammals: helper T cells and killer T cells. The role of killer T cells is to destroy infected cells, while helper T cells coordinate their attacks. Generally speaking, T cell differentiation are as follows: naive T cells express low levels of CD62 and CCR7; effector T cells express high levels of CD62 but low levels of CCR7; effect memory T cells express high levels of CD62 and a certain level of CCR7; central memory T cells express high levels of CD62 and CCR7. In 1970s, the existence of the different T cell populations was confirmed in bony fishes for the first time (110, 111). Sea bass currently constitute the only marine organism with a specific anti-T cell marker (DLT15) (16). In the present study, the clusters with the expressions of *CD3* (42, 43), *LCK* (44) and *ZAP70* (45) were clustered into the T cell population. The TCR/CD3 complex of traditional T cells recognizes specific antigens that bind to MHC and initiates the T cell activation (42, 43). Tyrosine kinases such as Lck and ZAP70 participate in the downstream T cell activation signaling pathway after the CD3ϵ chain (44, 45) of the T cell receptor is engaged. Furthermore, T cells were sub-clustered into specific 3 subsets (data not shown). However, we failed to characterize these clusters based on known T cell markers such as *CCR7* and *CD62L*. We suggest that further study collecting samples at different infection time points may improve this unsatisfactory situation.

## Conclusion

In conclusion, we carried out scRNA-seq analysis of the entire spleen from *E. coioides*-a teleost whose genome has not yet been published, and analyzed the teleost’s cell composition. Our research will provide new insights to better understand the biological characteristics, development and function of immune cell populations of teleost. The usage of the full-length transcriptome as a reference, offers a reliable and improved alternative for scRNA-seq data analysis in teleost when no whole genome is available.

## Data Availability Statement

The datasets presented in this study can be found in online repositories. The names of the repository/repositories and accession number(s) can be found in the article/**Supplementary Material**.

## Ethics Statement

The animal study was reviewed and approved by The Animal Ethics Committee of Jimei University.

## Author Contributions

QY, LH, and YQ conceived the experiments. All authors assisted in the collection and interpretation of data. LH, QY, and H-PG wrote the manuscript. All authors contributed to the article and approved the submitted version.

## Funding

This work was supported by the Natural Science Foundation of Fujian Province (No. 2019J06020 and 2019J01695); the National Natural Science Foundation of China under contract No.41502319; the Innovation Driven Development Foundation of Guangxi (No. AD19245135 and AD19245161); the Doctoral Research Startup Fund of the Fourth Institute of Oceanography, Ministry of Natural Resources (No. 201803 and 201806); and the Fund of Hainan Provincial Key Laboratory of Tropical Maricultural Technologies (No. TMTOF202104).

## Conflict of Interest

The authors declare that the research was conducted in the absence of any commercial or financial relationships that could be construed as a potential conflict of interest.

## Publisher’s Note

All claims expressed in this article are solely those of the authors and do not necessarily represent those of their affiliated organizations, or those of the publisher, the editors and the reviewers. Any product that may be evaluated in this article, or claim that may be made by its manufacturer, is not guaranteed or endorsed by the publisher.

## Supplementary Material

Supplementary Figure 1 Prediction of the lncRNA and alternative splicing events. **(A)** Venn diagram of lncRNA prediction results. **(B)** Statistic results of alternative splicing (AS) events. A3, alternative 3’ splice site; A5, alternative 5’ splice site; AF, alternative first exon; AL, alternative last exon; MX, mutually exclusive exon; RI, retained intron; SE, skipped exon.

Supplementary Figure 2 Mapping results of cell clustering based on the whole genome of *E. lanceolatus* back to the results of the full-length transcriptome. The tSNE nonlinear clustering was used to visualize the results.

Supplementary Figure 3 Visualization of the classification results of *E. coioides* spleen cell populations based on the full-length transcriptome **(A)** and *E. lanceolatus* whole genome **(B)** with UMAP.

Supplementary Table 1 Ordering Genes.

Supplementary Table 2 Detailed gene expression results of each cell population based on the E. lanceolatus genome.
