# Supplementary figures and images for: Full-Length Transcriptome: A Reliable Alternative for Single-Cell RNA-Seq Analysis in the Spleen of Teleost Without Reference Genome
Source: Front Immunol. 2021 Sep 27;12:737332. doi: 10.3389/fimmu.2021.737332 (PMC8502891; doi:10.3389/fimmu.2021.737332)

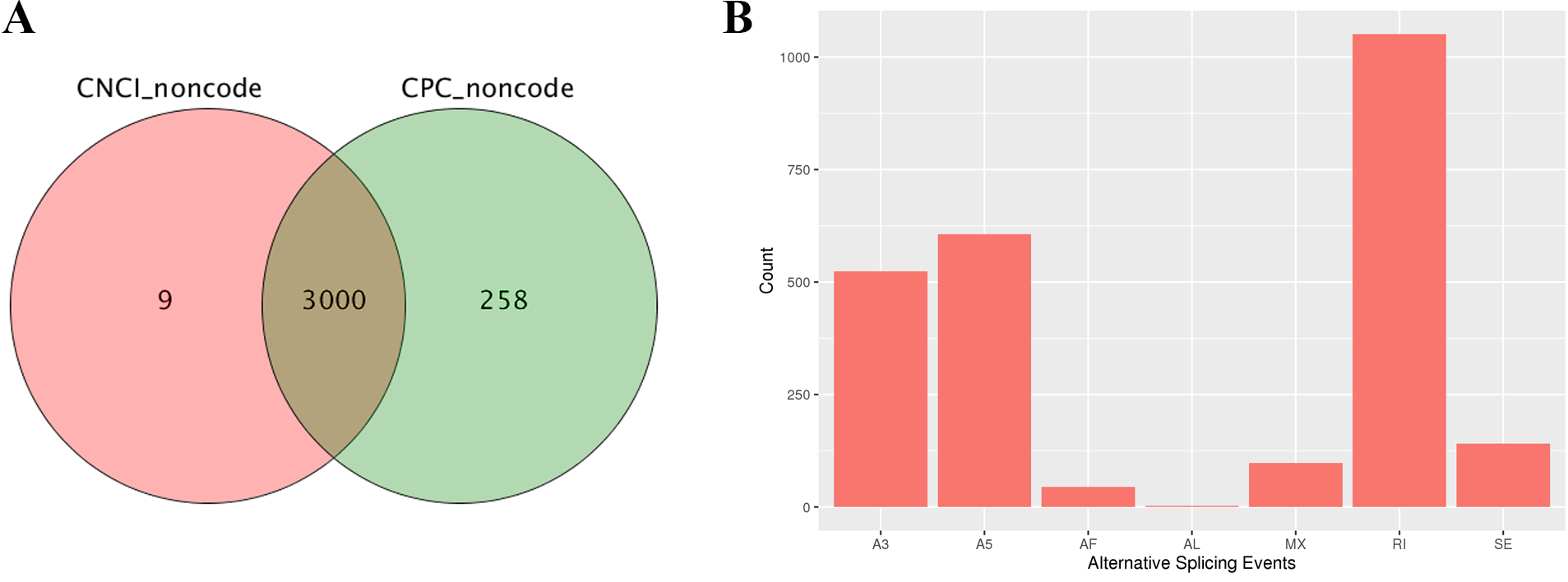

Supplement: Supplementary Figure 1 — Prediction of the lncRNA and alternative splicing events. (A) Venn diagram of lncRNA prediction results. (B) Statistic results of alternative splicing (AS) events. A3, alternative 3’ splice site; A5, alternative 5’ splice site; AF, alternative first exon; AL, alternative last exon; MX, mutually exclusive exon; RI, retained intron; SE, skipped exon. [file Image_1.tif]

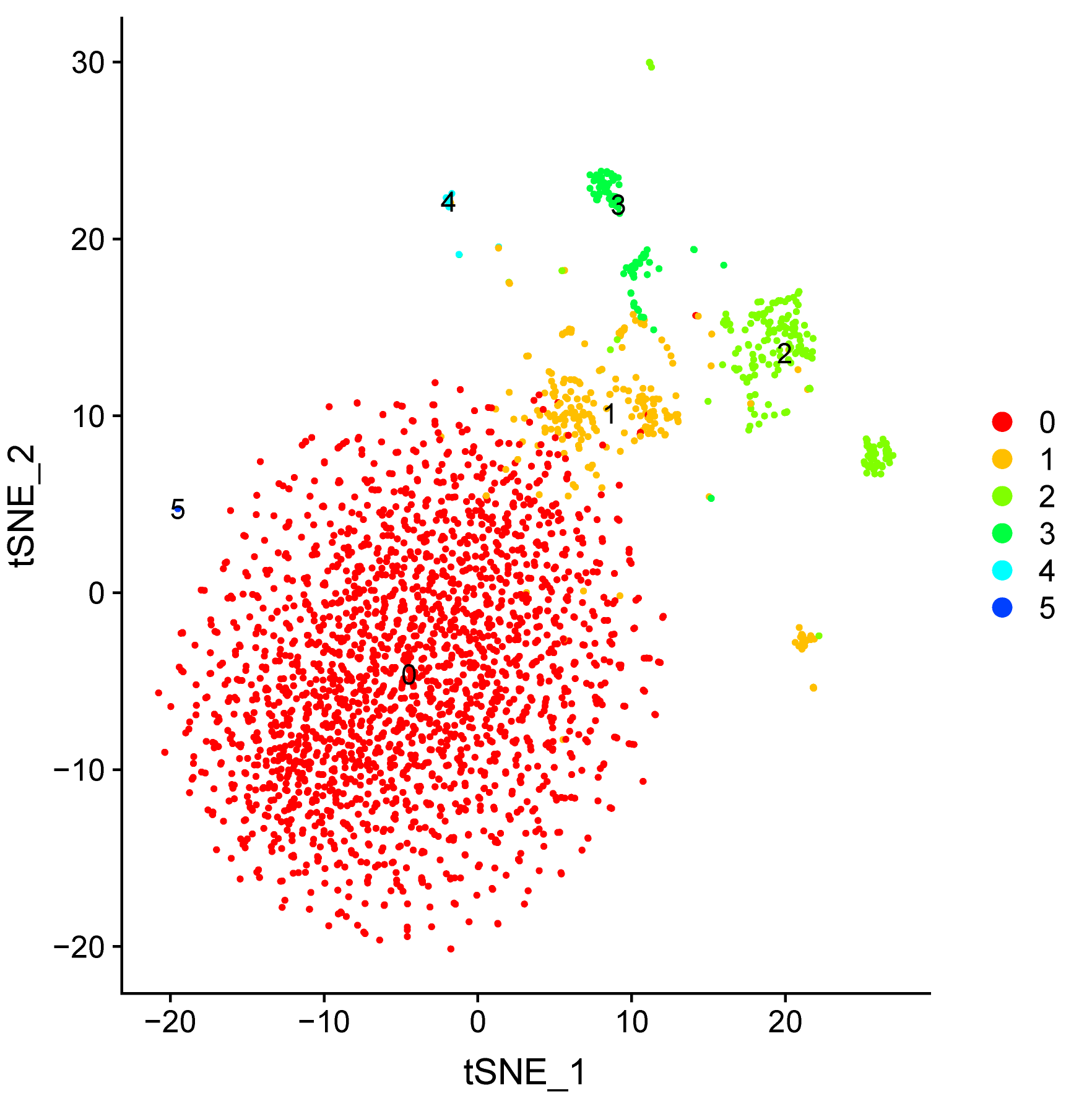

Supplement: Supplementary Figure 2 — Mapping results of cell clustering based on the whole genome of E. lanceolatus back to the results of the full-length transcriptome. The tSNE nonlinear clustering was used to visualize the results. [file Image_2.tif]

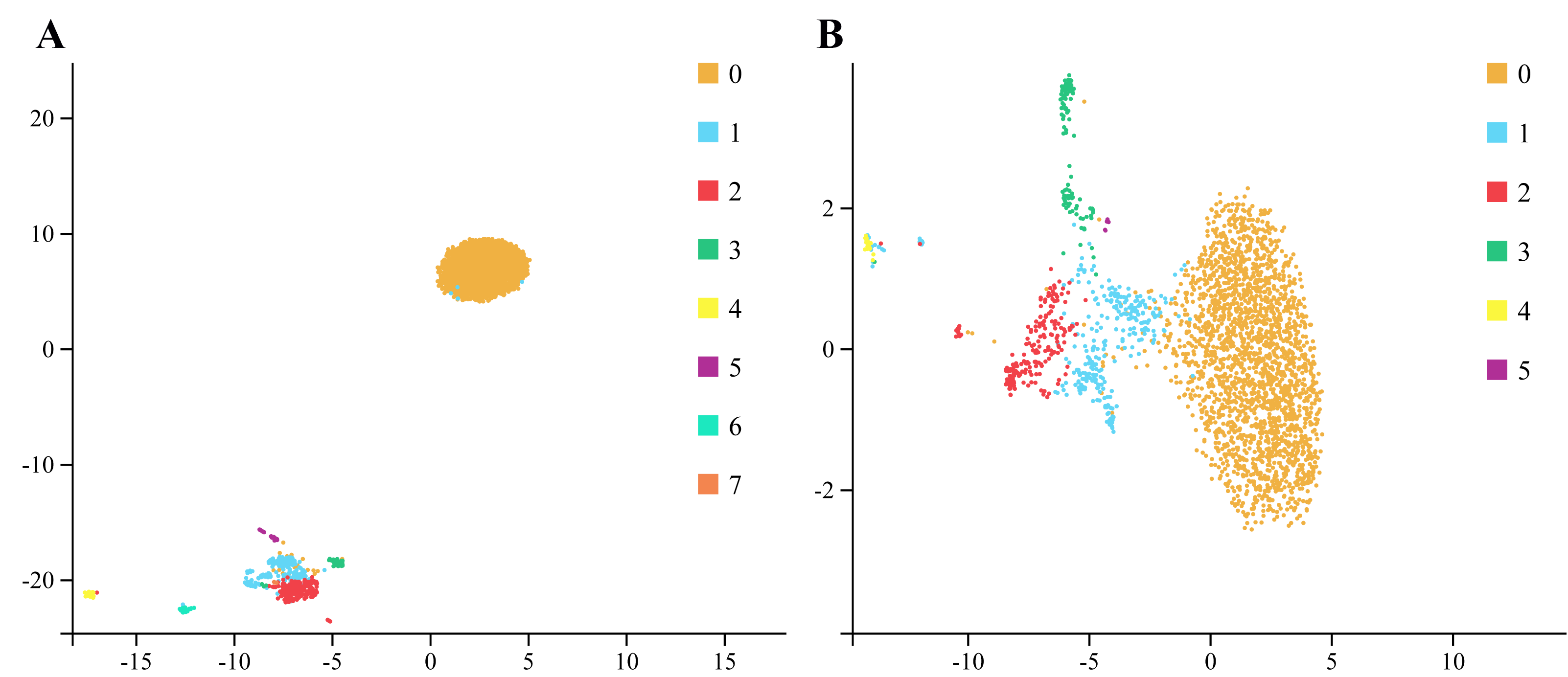

Supplement: Supplementary Figure 3 — Visualization of the classification results of E. coioides spleen cell populations based on the full-length transcriptome (A) and E. lanceolatus whole genome (B) with UMAP. [file Image_3.tif]
